# A 640 nA *I*_Q_ Output-Capacitor-Less Low Dropout (LDO) Regulator with Sub-Threshold Slew-Rate Enhancement for Narrow Band Internet of Things (NB-IoT) Applications

**DOI:** 10.3390/mi15081019

**Published:** 2024-08-09

**Authors:** Yuxin Zhang, Jueping Cai, Jizhang Chen, Yixin Yin

**Affiliations:** Shaanxi Key Lab of Integrated Circuits and Systems, School of Microelectronics, Xidian University, Xi’an 710071, China; yuxinzhang@stu.xidian.edu.cn (Y.Z.); jz_chen@stu.xidian.edu.cn (J.C.); 20009100987@stu.xidian.edu.cn (Y.Y.)

**Keywords:** capacitor-less low dropout regulator, low power, sub-threshold, slew-rate enhancement, narrow band Internet of Things

## Abstract

An ultra-low quiescent current output-capacitor-less low dropout (OCL-LDO) regulator for power-sensitive applications is proposed in this paper. To improve the gain of the OCL-LDO feedback loop, the error amplifier employs a combination of a cross-coupled input stage for boosting the equivalent input transconductance and a negative resistance technique to improve the gain. Meanwhile, in order to address the issue of transient response of the ultra-low quiescent current OCL-LDO, a sub-threshold slew-rate enhancement circuit is proposed in this paper, which consists of a transient signal input stage and a slew-rate current increase branch. The proposed OCL-LDO is fabricated in a 0.18 μm CMOS process with an effective area of 0.049 mm^2^. According to the measurement results, the proposed OCL-LDO has a maximum load current of 100 mA and a minimum quiescent current of 640 nA at an input voltage of 1.2 V and an output voltage of 1 V. The overshoot and undershoot voltages are 197 mV and 201 mV, respectively, and the PSR of the OCL-LDO is −72.4 dB at 1 kHz when the load current is 100 μA. In addition, the OCL-LDO has a load regulation of 7.6 μV/mA and a line regulation of 0.87 mV/V.

## 1. Introduction

**Figure 1:**
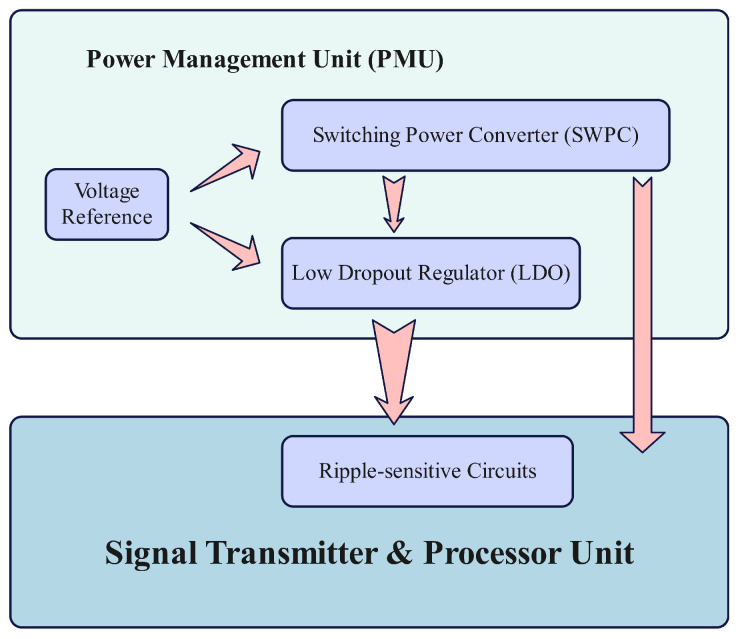
The classic composition of an NB-IoT application.

Narrow band Internet of Things (NB-IoT), a critical technology focusing on low-power applications within the IoT field, exhibits great potential for applications in precision medicine, smart transportation, and environmental monitoring, among others [1,2,3,4]. Nevertheless, the challenge of power management units (PMUs) for NB-IoT applications has become increasingly difficult because of the nature of NB-IoT applications in which batteries are difficult to replace frequently. Consequently, it is necessary to reduce the quiescent currents of critical circuits within PMUs, which leads to extended battery life [5,6,7]. Low dropout regulator (LDO) owing to its high ripple immunity is a power management unit (PMU) topology that is placed after the switching power converter (SWPC) to provide a reliable power supply for ripple sensitive circuits as shown in Figure 1 [8,9,10]. Furthermore, in order to improve the current efficiency under ultra-low loads of the LDOs, their quiescent current is supposed to be low (≤1 μA).

A number of works have been presented on the circuit design of ultra-low quiescent current LDOs, categorized into conventional LDOs with off-chip μF-class capacitors [11,12,13] and highly integrated output-capacitor-less LDOs [14,15,16,17]. Although off-chip μF-level capacitors are beneficial for system stability and transient response, etc., OCL-LDOs have become a more preferred choice among them, as NB-IoT applications increasingly demand higher levels of integration [18,19,20]. With the development of CMOS process towards the nanoscale, the current consumption of digital cells within NB-IoT applications is able to vary between close to zero and a maximum value in a short time [21,22,23,24]. Thus, the low dropout regulator is expected to have a favorable transient response to counteract large fluctuations in load current over a short time. Since there is no bulky off-chip capacitor to store charge for improving transient response, and the ultra-low quiescent current results in the reduction in the loop bandwidth and an insufficient swing rate required at the gate of the power-FET, the transient response of the ultra-low quiescent current OCL-LDOs is in demand for improvement.

Several nA-level quiescent current OCL-LDO circuit structures have already been presented in previous work. Huang et al. proposed an OCL-LDO with a quiescent current of only 10 nA [14], which utilizes an adaptive biasing scheme to reduce the quiescent current of the OCL-LDO when it is under ultra-low load. Meanwhile, a hybrid of analog and digital transient enhancement has been proposed to improve the transient response of this OCL-LDO. The bias current of the comparator used in the transient enhancement circuit of [14] is only 1.5 nA, so the impact of the delay of the comparator on the recovery time of the transient response is not negligible. In addition to this, the hybrid compensation approach increases the complexity of the circuit design and chip area. Zhao et al. proposed a feed-forward transient enhancement circuit, which couples the variation of the output voltage directly to the current-controlled oscillator via a capacitor for fast transient response [25]. However, the measurement results of its load regulation and line regulation show that this OCL-LDO experiences large variations with changes in the load current or input voltage. The transient enhancement circuits proposed in [14,25] both contain digital circuit components; although they cause no increase in the quiescent current of the OCL-LDO, they demand a high dynamic power consumption of the circuit. An OCL-LDO with a supply voltage of 0.6 V and a quiescent current of only 220 nA was proposed by Zhang et al. which utilizes bulk modulation to reduce the power of the circuit, but its maximum load current is only 10 mA, making it difficult to satisfy the current requirements of high-performance operating states for NB-IoT applications [26]. Tang et al. suggested an embedded slew-rate enhancement circuit for improving the transient response of the OCL-LDO, but the quiescent current of this module is 3.6 μA, which is unsuitable for low-power situations [27].

An ultra-low quiescent current output-capacitor-less low dropout voltage regulator is proposed in this paper. With the aim of improving the accuracy of the output voltage of the OCL-LDO, i.e., increasing the gain of the feedback loop without applying a three-stage amplification structure, the error amplifier within this OCL-LDO employs a cross-coupled input stage to increase the equivalent transconductance, and utilizes a negative-resistance technique to increase the gain of the error amplifier. The improvement in loop gain simultaneously improves the ripple immunity, that is to say, the power supply rejection (PSR) of this OCL-LDO. In response to the problem of weak transient response due to ultra-low quiescent current, this paper presents a sub-threshold CMOS-based slew-rate enhancement circuit that incorporates a transient signal introduction and a slew rate current increase branch. An equivalent GΩ-level resistor of the MOSFET connected to the gate and the source is used for the sub-threshold CMOS slew-rate enhancement circuit in order to minimize the area of the chip, and the effect of transient voltage variations on the main loop is also eliminated. The OCL-LDO is fabricated in the TSMC 0.18 μm CMOS process with an area of 0.049 mm^2^. Measurement results show that the minimum quiescent current is 640 nA at an input voltage of 1.2 V and an output voltage of 1 V of the proposed OCL-LDO. The maximum load current of the proposed OCL-LDO is 100 mA since the size of the power transistor is 12 mm/0.2 μm. Although the pole at the gate of the power transistor is close to the output pole due to the large size of power-FET, the OCL-LDO remains stable at a load current of 1 μA because of the Miller compensation capacitor. Meanwhile, with a certain power transistor size, the wider the bias voltage range, the greater the load current range, i.e., the higher the maximum load current. In order to increase the voltage range at the gate of the power transistor, i.e., the bias voltage of power-FET, the error amplifier employs a Class-AB output stage structure, which has a rail-to-rail output range. With the aid of the sub-threshold CMOS slew-rate enhancement circuit, the overshoot and undershoot voltages of this OCL-LDO are 194 mV and 201 mV, respectively, when the load current is switched between 100 μA and 100 mA.

The remainder of this paper is organized as follows. Section 2 presents the OCL-LDO design proposed in this paper and its stability analysis. Section 3 illustrates the circuit implementation and design considerations in detail. Section 4 shows the measurement results, and Section 5 provides a summary of the measurement results and a comparison with prior works. Section 6 gives the conclusion of this paper.

## 2. Architecture and Stability Analysis of Proposed OCL-LDO

The OCL-LDO is required to be designed with ultra-low quiescent current in NB-IoT applications. As a negative feedback control system, the OCL-LDO firstly requires its loop gain and phase to be analyzed to ensure the stability of the system. Meanwhile, a circuit design with ultra-low quiescent current deteriorates its transient response, which is not able to satisfy the demand of rapidly switching operating states in IoT applications. Hence, a sub-threshold CMOS-based slew-rate enhancement circuit is proposed to optimize the transient response of the OCL-LDO, and the sub-threshold CMOS-based circuit is designed with nA-level quiescent currents, which will not burden the OCL-LDO with excessive quiescent currents as a whole.

### 2.1. Architecture of Proposed OCL-LDO

**Figure 2:**
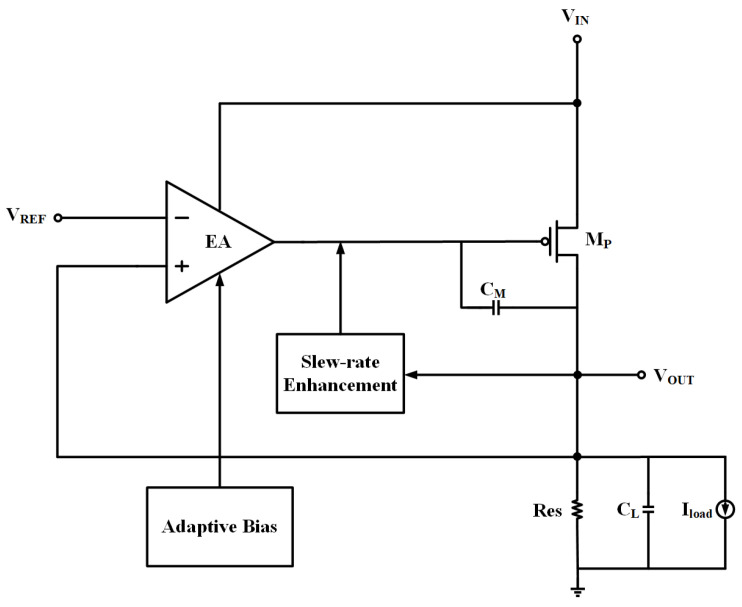
Structure of proposed OCL-LDO.

In order to minimize the quiescent current of the output-capacitor-less low-dropout linear regulator (OCL-LDO), the number of current branches in the system should be fewer, so the proposed OCL-LDO in this paper is composed of a two-stage amplification structure, which is shown in Figure 2. The two-stage amplification structure composed of an error amplifier and a power-FET is used to provide a stable voltage output, where the first stage of amplification, i.e., the error amplifier, compares the output voltage with the reference voltage and provides an error signal, and then the output of the error amplifier is connected to the gate of the power-FET because the output voltage is fixed and the load current varies so the gate voltage needs to have a wide range of variation. For this reason, the Class-AB output stage is selected so that the error amplifier has a rail-to-rail output range. In order to make the current efficiency at ultra-low load current and the OCL-LDO performance at heavy load excellent at the same time, the system employs an adaptive biasing scheme. Furthermore, there is a sub-threshold CMOS slew-rate enhancement circuit embedded in the OCL-LDO, which effectively improves the transient response of the OCL-LDO, and its nano-ampere quiescent current does not make the overall quiescent current of the OCL-LDO exceed 1 μA.

### 2.2. Stability Analysis

**Figure 3:**
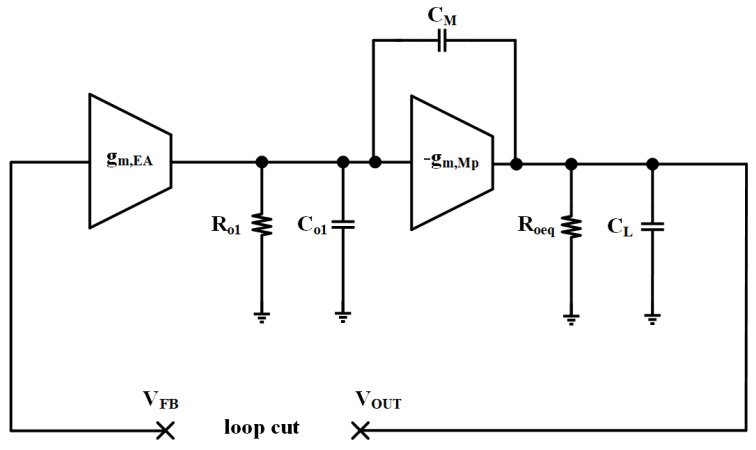
Small-signal model of proposed OCL-LDO without slew-rate enhancement circuit.

The sub-threshold CMOS slew-rate enhancement circuit is implemented in the proposed LDO. This circuit detects transient changes in the output voltage and quickly provides feedback to the gate of the power field effect transistor. The small-signal model of the proposed OCL-LDO without considering the sub-threshold slew-rate enhancement circuit is shown in Figure 3.

Based on the analysis, the small-signal transfer function of the LDO when there is no compensation network may be represented as follows:

(1) $$T\left(s\right)\approx \frac{-g_{m,EA}g_{m,Mp}R_{o1}R_{oeq}}{\left(1+sR_{oeq}C_{L}\right)\left(1+sR_{o1}C_{G}\right)}$$

where $g_{m,EA}$ and $g_{m,Mp}$ denote the transconductance of the error amplifier and the power transistor, respectively, $R_{o1}$ and $R_{oeq}$ denote the equivalent output resistance of the error amplifier and the equivalent output resistance of the OCL-LDO, respectively, $C_{L}$ is the load capacitance, and $C_{G}$ is the gate parasitic capacitance of power-FET. In the absence of Miller compensation, the transfer function of the OCL-LDO loop displays two poles that are quite near each other in some cases. This closeness leads in a narrow phase margin, which may not fulfill the stability criteria for the system. To address this, the addition of a Miller compensation capacitor, $C_{M}$, becomes necessary. The Miller compensation capacitor is used to generate isolation between the primary and secondary poles of the transfer function. By connecting the $C_{M}$ between the output of the error amplifier and the input of the power transistor, the output of the error amplifier is able to be fixed as the primary pole, which is reduced to a large extent compared to the initial error amplifier output pole, while the $C_{M}$ may successfully boost the size of the secondary pole, thus enlarging the phase margin. At this stage, the loop transfer function of the OCL-LDO may be stated as:

(2) $$T\left(s\right)\approx \frac{-g_{m,EA}g_{m,Mp}R_{o1}R_{oeq}}{\left(1+sC_{L}/g_{mp}\right)\left(1+g_{mp}R_{oeq}C_{M}R_{o1}\right)}$$

in which $g_{mp}$/$C_{L}$ could be used to approximate the secondary point and 1/$g_{mp}$$R_{oeq}$$C_{M}$$R_{o1}$ to approximate the primary pole. Regardless of the load current modifications, the secondary point is much greater than the primary pole after Miller compensation.

**Figure 4:**
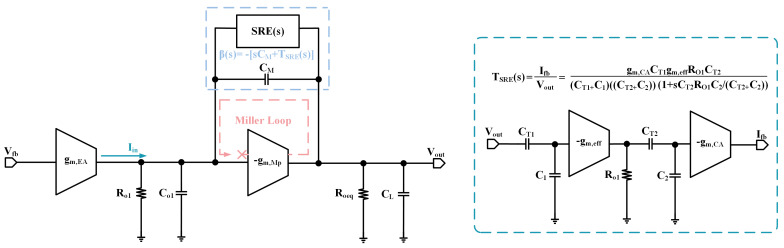
Small-signal model of proposed OCL-LDO with slew-rate enhancement circuit.

The sub-threshold slew-rate enhancement circuit is designed to sense changes in the output voltage and transfer the resulting signal variation to the gate of the power-FET through a fast feedback loop, which then negatively feeds back the output voltage. When considering the effect of the sub-threshold slew-rate enhancement circuit on the main loop, its small-signal model can be represented as shown in Figure 4. The connection is the same as the Miller capacitor and the transfer function is expressed as:

(3) $$T_{SRE}\left(s\right)=\frac{I_{fb}}{V_{out}}=\frac{1}{\left(C_{1}+C_{T1}\right)\left(C_{2}+C_{T2}\right)}\times \frac{C_{T1}g_{m,eff}R_{O1}C_{T2}g_{m,CA}}{\left(1+sR_{O1}C_{T2}C_{2}/\left(C_{T2}+C_{2}\right)\right)}$$

in which $C_{T1}$ and $C_{T2}$ are couple capacitors to the transient signal input stage and slew-rate enhancement branch. $C_{1}$ and $C_{2}$ are the input parasitic capacitance. $R_{O1}$ is the output resistance of the transient signal input stage. $g_{m,eff}$ and $g_{m,CA}$ are equivalence transconductance of the transient signal input stage and slew-rate current increase branch.

A design-oriented analysis (DOA) is adopted in this paper to avoid the complexity of transfer functions and to provide a more intuitive understanding of the impact of the zero poles of the OCL-LDO on the stability of this system [28,29,30]. The slew-rate enhancement circuit and $C_{M}$ together form the Miller loop:

(4) $$\begin{array}{c} \beta \left(s\right)=-\left[sC_{C}+T_{SRE}\left(s\right)\right]=-\left[sC_{C}+\frac{A}{\left(1+sR_{O1}C_{T2}C_{2}/\left(C_{T2}+C_{2}\right)\right)}\right] \end{array}$$

where *A* is the coefficient and $C_{C}$ is the combination of the $C_{M}$ and $C_{gd}$ parasitic capacitance of power-FET:

(5) $$\begin{array}{c} A=\frac{C_{T1}g_{m,eff}R_{O1}C_{T2}g_{m,CA}}{\left(C_{1}+C_{T1}\right)\left(C_{2}+C_{T2}\right)} \end{array}$$

According to DOA, the transfer function of Miller loop is obtained and can be expressed as:

(6) $$\begin{array}{cccc} T_{ML}\left(s\right) & =\beta \left(s\right)H\left(s\right) & \approx \left[sC_{C}+\frac{A}{\left(1+sR_{O1}C_{T2}C_{2}/\left(C_{T2}+C_{2}\right)\right)}\right] & \cdot \left[\frac{g_{mp}R_{O1}R_{oeq}}{\left(1+sR_{oeq}C_{L}\right)\left[1+sg_{mp}R_{O1}R_{oeq}\left(C_{O1}+C_{C}\right)\right]}\right] \end{array}$$

where H(s) is the open loop transfer function of transimpedance amplifier. By simplifying the above equation, it is obtained that:

(7) $$\begin{array}{c} T_{ML}\left(s\right)\approx \frac{sC_{C}g_{mp}R_{O1}R_{oeq}}{A}\;\left[\frac{1}{\left(1+sR_{oeq}C_{L}\right)\left[1+sg_{mp}R_{O1}R_{oeq}\left(C_{O1}+C_{C}\right)\right]}\right] \end{array}$$

As a result, the pole positions obtained from Equations (2) and (7) are the same, the primary pole $p_{1}$ is located at the gate of the power-FET, and the secondary pole is located at the output voltage of the OCL-LDO:

(8) $$p_{1}=\frac{1}{g_{mp}R_{O1}R_{oeq}\left(C_{o1}+C_{C}\right)},p_{2}=\frac{1}{R_{oeq}C_{L}}$$

**Figure 5:**
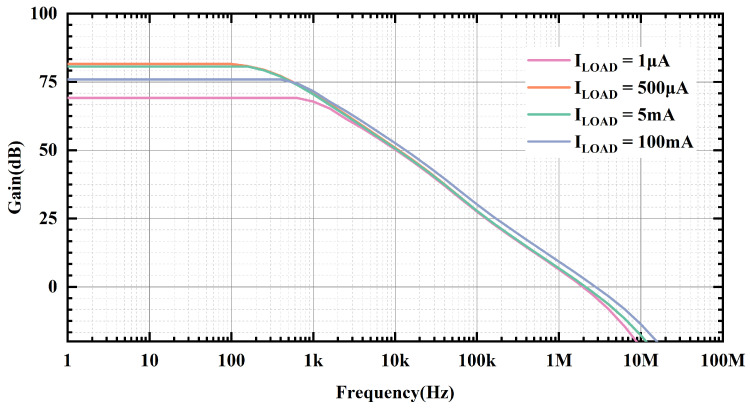
Simulated loop gain of proposed OCL-LDO under different load currents when $C_{L}$ is 100 pF and temperature is room temperature.

**Figure 6:**
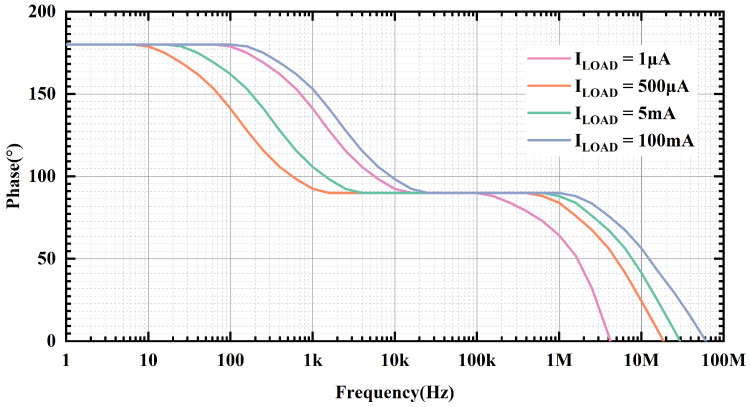
Simulated loop phase of proposed OCL-LDO under different load currents when $C_{L}$ is 100 pF and temperature is room temperature.

**Figure 7:**
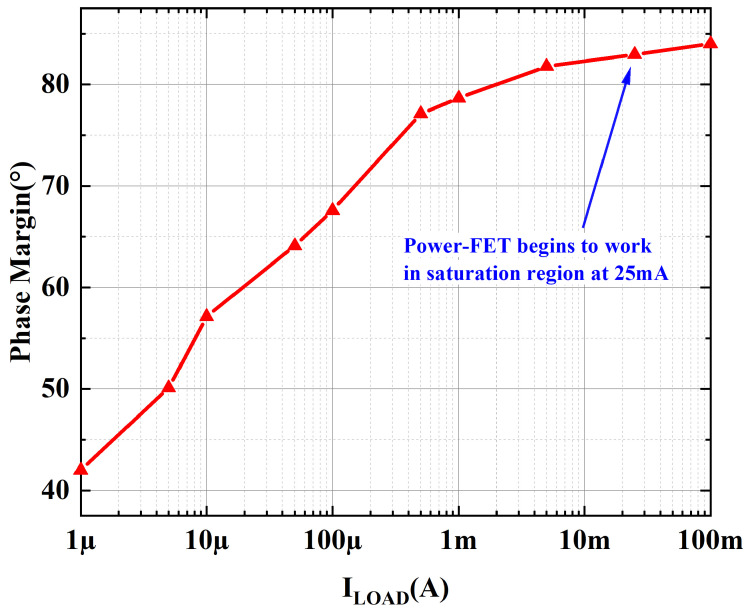
Simulated phase margin vs. load current.

Figure 5 and Figure 6 provide the loop gain and phase simulation results at room temperature, input voltage of 1.2 V and output voltage of 1 V with different load currents, respectively. At ultra-low loads, its secondary poles are close to the main poles. As the load current increases, the output equivalent resistance decreases, so the secondary pole is far away from the main pole, and the phase margin becomes more favorable. Figure 7 presents the phase margin at different load currents when the load capacitance is 100 pF, and the variation trend of the PM is consistent with the previous analysis. The minimum phase margin (PM) is 42.5° when the load current is 1 μA. And when the load current is greater than 25 mA, the power-FET begins to operate in the saturation region, at which time the transconductance of the power transistor is greater than the $g_{m}$ in the sub-threshold region, and the frequency where the secondary poles are located is greater than the GBW of the OCL-LDO; therefore, the phase margin is better during heavy loads.

## 3. Circuit Implementation and Design Considerations

The OCL-LDO proposed in this paper is capable of operating at nA-level quiescent currents as well as with good transient response characteristics. Its slew-rate enhancement circuit is based on sub-threshold CMOS operation, so even with the addition of a circuit module, the quiescent current of OCL-LDO is still lower than 1 μA.

### 3.1. Overall Circuit of Proposed OCL-LDO

**Figure 8:**
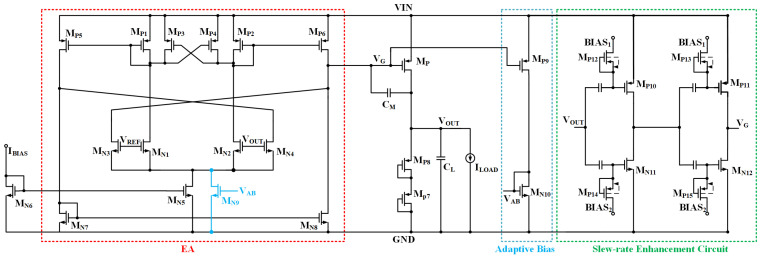
Overall circuit of proposed OCL-LDO.

The concrete circuit of the OCL-LDO proposed in this paper is shown in Figure 8, which includes three parts: the main loop composed of error amplifiers and power transistors, the adaptive biasing scheme that adjusts the bias current of the OCL-LDO according to the load current, and the sub-threshold CMOS slew-rate enhancement circuit.

To enhance the accuracy of the output voltage so that it is not subject to large fluctuations with changes in the load and input voltage, the gain of the error amplifier is required to be high. And in order to reduce the quiescent current and at the same time reduce the design complexity, the OCL-LDO proposed in this paper is a two-stage structure, which improves the gain of the error amplifier by embedding the cross-coupled input transistors and negative resistance technique. A sub-threshold slew rate enhancement circuit is proposed to alleviate the problem of poor transient response due to ultra-low quiescent current. It includes an RC circuit for coupling the transient changes in the output voltage, i.e., a transient signal input stage, and then the transient changes in the output signal of this module circuit are transferred to the slew-rate current increase branch through capacitive coupling. In this regard, the resistors in the sub-threshold slew-rate enhancement circuit are all implemented by gate and source connected (GSC) MOSFETs, which could be equivalent to a GΩ-level resistor. This makes the transient voltage changes not affect the main loop and reduces the recovery time.

### 3.2. High-Gain Error Amplifier

In order to minimize the impact of input voltage and load current variations on the output voltage of the proposed OCL-LDO, it is desirable to increase the gain of the error amplifier in the main loop, but the three-stage amplification structure imposes additional quiescent current consumption, while the design complexity of the compensation circuit is increased.

The addition of a negative resistance and cross-coupled input stage requires no additional quiescent current consumption and is also effective in increasing the gain of the error amplifier. The gain of the error amplifier when there is no negative resistance and cross-coupled inputs stage is:

(9) $$Gain_{DC}=G_{m}\times \left(R_{on}\|R_{op}\right)$$

in which $G_{m}$ is the equivalence transconductance of the error amplifier, and $R_{on}$ and $R_{op}$ are the on-resistance of $M_{N8}$ and $M_{P6}$. When there is a negative resistance in the amplifier, Equation (9) can be expressed as:

(10) $$Gain_{DC}=G_{m}\times \Delta \frac{1}{g_{m}}\times g_{m,P6}\times \left(R_{on}\|R_{op}\right)$$

where Δ$g_{m}$ is the difference in transconductance, and $g_{m,P6}$ is the transconductance of $M_{P6}$. The cross-coupled input stage makes the equivalent input transconductance of the error amplifier twice as large, and the on-resistance of $M_{N3}$ can be seen as a part of the output impedance of the error amplifier, where $M_{N9}$ and $M_{N5}$ are connected in parallel and then connected in series with $M_{N3}$, so the impedance as seen from the drain of $M_{N3}$ is:

(11) $$R_{out}=\left(1+g_{m,N3}r_{o,N3}\right)\left(r_{o,N5}\|r_{o,N9}\right)$$

Thus, the DC gain of the error amplifier at this point may be expressed as:

(12) $$Gain_{DC}=\left[G_{m}+G_{m}\times \Delta \frac{1}{g_{m}}\times g_{m,P6}\right]\times \left(R_{on}\left\|R_{op}\right\|R_{out}\right)$$

Since $R_{out}$ is far greater than $R_{on}$ and $R_{op}$, the gain of the error amplifier is simplified to:

(13) $$Gain_{DC}\approx \left[G_{m}+G_{m}\times \Delta \frac{1}{g_{m}}\times g_{m,P6}\right]\times \left(R_{on}\|R_{op}\right)$$

**Figure 9:**
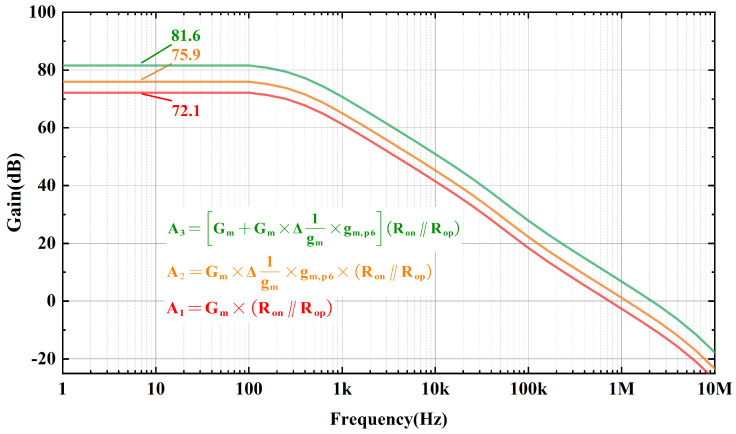
Loop gain of proposed utilizes different error amplifier.

Comparison of simulation results for loop gain at a load current of 500 μA is shown in Figure 9, where it is observed that the addition of negative resistance and the utilization of a cross-coupled input stage effectively improves the gain of the error amplifier.

According to Equations (9), (10) and (13), the DC gain of the error amplifier is calculated approximately, and by multiplying it with the gain of power-FET, the loop gain calculated results in Table 1 are obtained. As seen in Table 1, the calculated results compare well with the simulated results, so the negative resistance and the cross-coupled input stage increase the loop gain of the OCL-LDO by boosting the DC gain of the error amplifier.

### 3.3. Sub-Threshold CMOS Slew-Rate Enhancement Circuit

The ultra-low quiescent current circuit design makes the loop bandwidth of the OCL-LDO decrease, while the small charge/discharge current required at the gate of the power transistor leads to inferior transient response of the OCL-LDO. Therefore, in order to optimize the transient response of the OCL-LDO proposed in this paper, the first approach is to utilize an adaptive biasing scheme, where the heavy load corresponds to the increase in the quiescent current with the increase in the load current when switching from a low load (which means the load current is 100 μA or below it) to a heavy load (which is a load current that is 50 mA or above it), and at this time, both the slew-rate and the loop bandwidth are improved, and thus the adaptive biasing scheme is able to effectively reduce the undershoot voltage and its recovery time.

**Figure 10:**
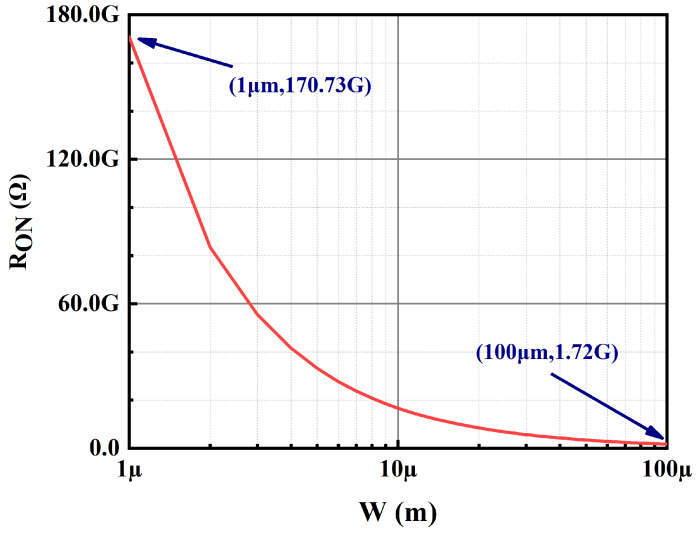
$R_{on}$ of GSC PMOS when L = 0.2 μm.

**Figure 11:**
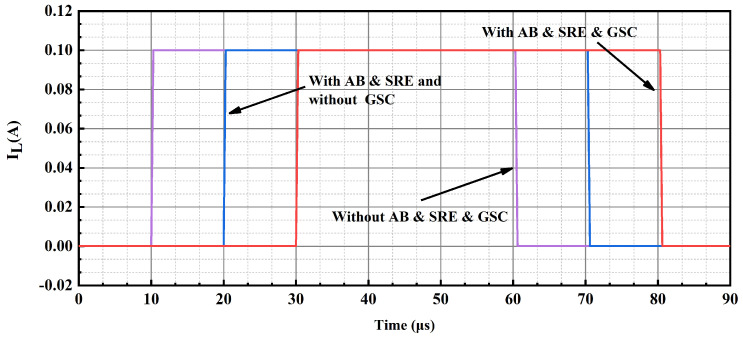
Load current changes between 100 μA and 100 mA in 500 ns.

**Figure 12:**
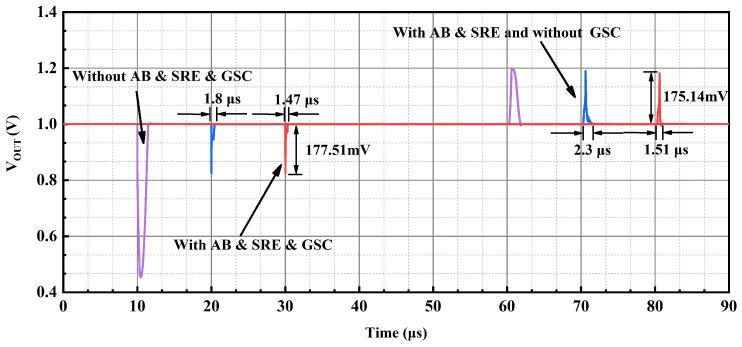
Simulation result of load transient response as $V_{\mathrm{IN}}$ = 1.2 V and $V_{\mathrm{OUT}}$ = 1 V.

In addition to this, a sub-threshold CMOS-based slew-rate enhancement circuit is proposed to further improve the transient response of the OCL-LDO. $M_{P12}$–$M_{P15}$ of the slew-rate enhancement are GSC MOSFET, which is employed to supply the bias voltage. The simulation results of $R_{on}$ versus the width for GSC PMOS with a length of 200 nm is given in Figure 10, where it is observed that the equivalent resistance of the GSC transistor is GΩ-level. Take the low load to heavy load switching as an example; at this time, the output voltage results in the undershoot voltage, and the capacitor couples the same trend to the inputs of $M_{N11}$ and $M_{P10}$ in Figure 8, resulting in the drain voltage of $M_{P10}$ and $M_{N11}$, i.e., the output voltage of the transient signal introduction stage increases, and the inputs of the slew-rate increasing branch sense through the capacitor the same change as the output of the previous stage, i.e., the gate voltages of $M_{N12}$ and $M_{P11}$ increase simultaneously, and at this time the gate voltage is discharged to make the output voltage reach stability quickly. An inverter-like structure is employed in this slew-rate enhancement circuit with a rail-to-rail output range, but its quiescent current is determined by the bias voltage, while its effect on loop stability has been analyzed above, and it has no impact on the primary and secondary poles of the feedback loop. The simulation conditions for switching load current within 500 ns are given in Figure 11 for an input voltage of 1.2 V and an output voltage of 1 V, and the simulation results shown in Figure 12 are obtained, which show that this sub-threshold slew-rate enhancement circuit could improve the transient response of the proposed OCL-LDO.

**Figure 13:**
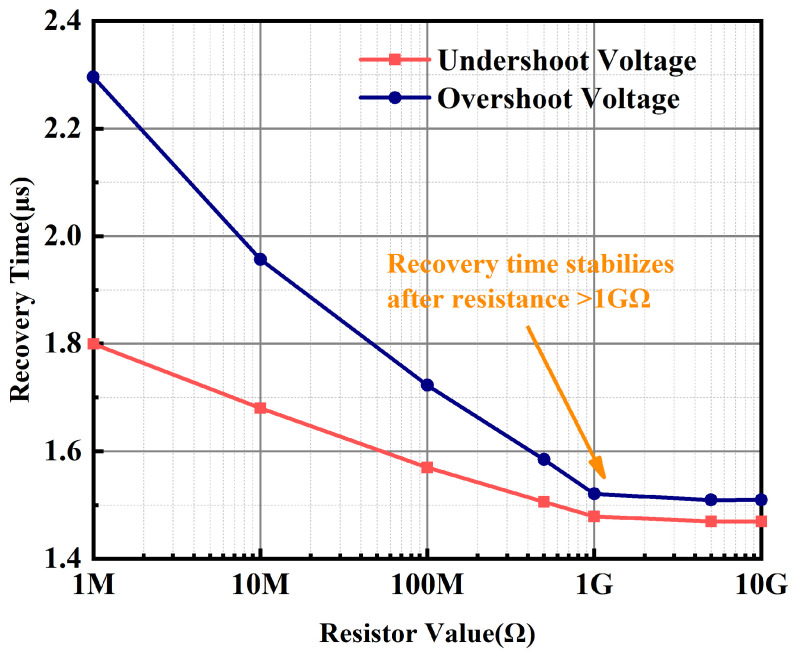
Simulation results of recovery time vs. resistor value.

The simulation results of the recovery times corresponding to the overshoot and undershoot voltages of OCL-LDOs as a function of the resistor value are shown in Figure 13. It is observed from Figure 13 that the recovery time of the transient voltage remains basically stable when the resistance is larger than 1 GΩ, that is, the variation in the transient voltage has no effect on the main loop. Because when the transistor width is 100 μm, the $R_{on}$ of GSC PMOS is 1.72 GΩ as shown in Figure 10, i.e., when the length of GSC PMOS is 200 nm, as long as the width of the transistor is less than 100 μm, it enables the main loop to be unaffected by the transient voltage change.

## 4. Measurement Results

**Figure 14:**
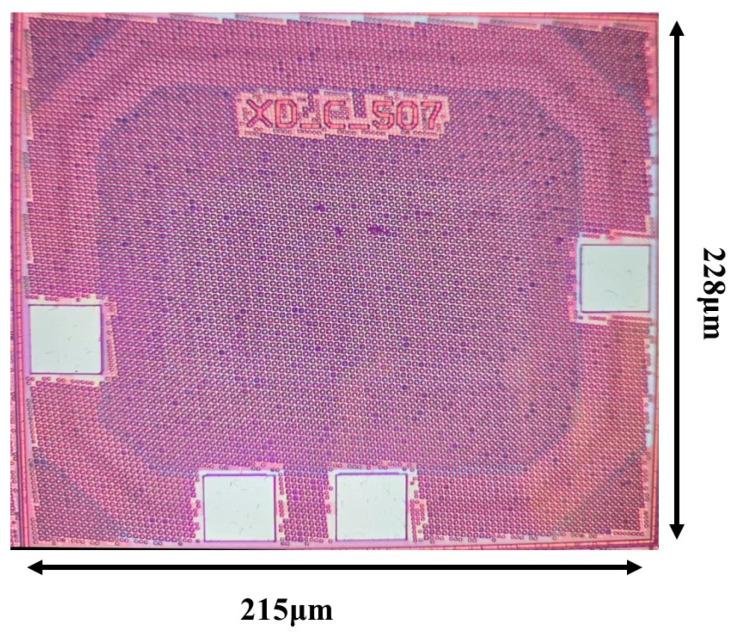
Photomicrograph of proposed OCL-LDO.

**Figure 15:**
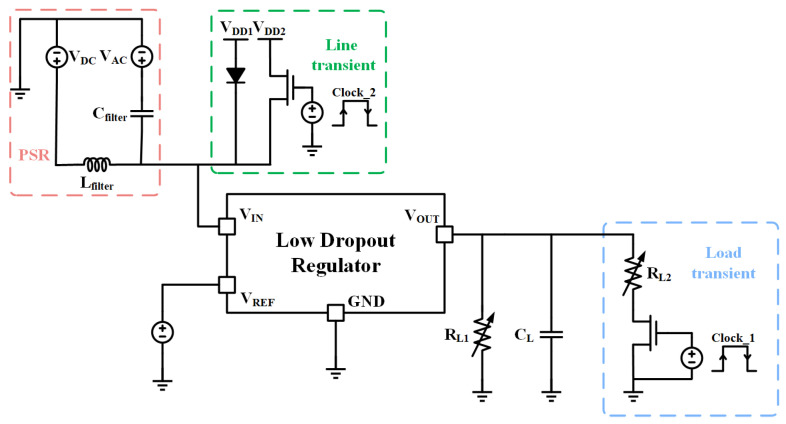
Schematic diagram of OCL-LDO measurements.

**Figure 16:**
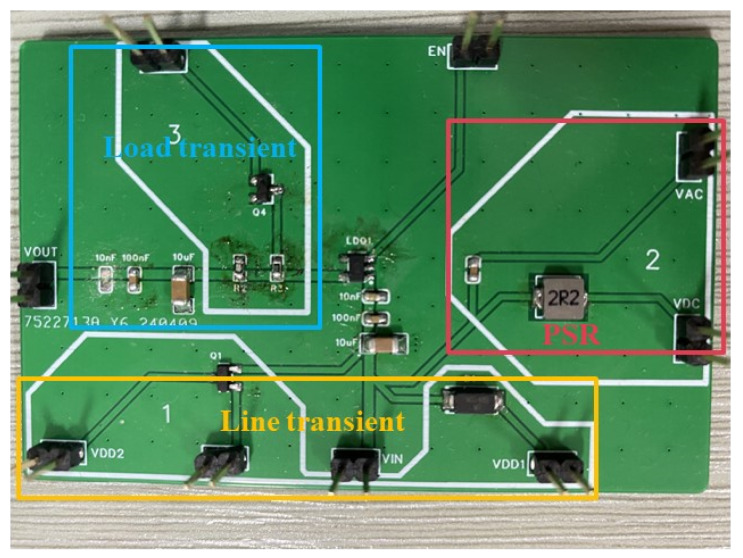
PCB board for the measurement of OCL-LDOs.

The ultra-low quiescent current OCL-LDO with the sub-threshold slew-rate enhancement circuit proposed in this paper is fabricated in a TSMC 0.18 μm process. The chip micrograph is shown in Figure 14, with an active area of 0.049 mm^2^ (228 μm × 215 μm). The dropout of this OCL-LDO is 200 mV, i.e., when the output is 1 V, the minimum input voltage is 1.2 V, at which point the OCL-LDO is capable of driving a load current range of 1 μA–100 mA. The minimum quiescent current is only 640 nA, and because of the adaptive biasing scheme, the quiescent current range of this OCL-LDO is 0.64–61.2 μA. The test circuit schematic of this OCL-LDO is given in Figure 15, and Figure 16 is the PCB test board corresponding to the test circuit schematic.

**Figure 17:**
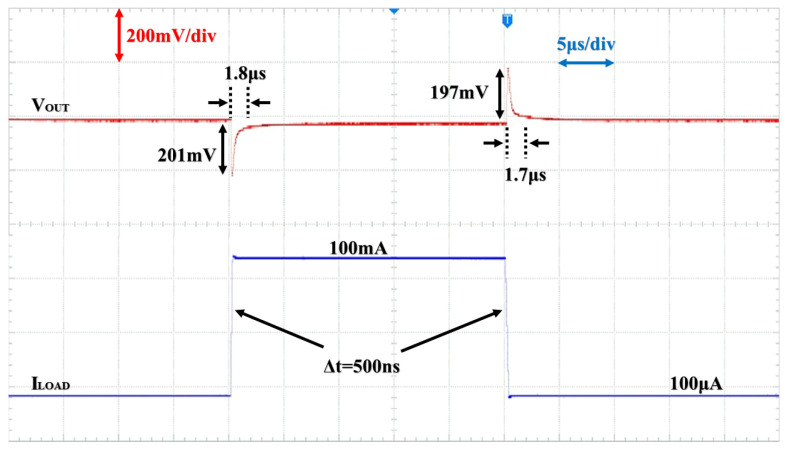
Load transient response as $V_{\mathrm{IN}}$ = 1.2 V and $V_{\mathrm{OUT}}$ = 1 V.

Figure 17 presents the load transient response of the OCL-LDO when the input voltage is 1.2 V and the output voltage is 1 V, where the load current switches between 100 μA and 100 mA load current within 500 ns. As seen in the figure, the overshoot voltage is 197 mV, corresponding to a recovery time of 1.7 μs. And when the load is changed from 100 μA to 100 mA, the undershoot voltage is 201 mV, with a recovery time of 1.8 μs. Compared with the simulation results in Figure 17, the overshoot and undershoot voltages of the measurement results are slightly increased, which is due to the parasitic capacitance at the output PAD.

**Figure 18:**
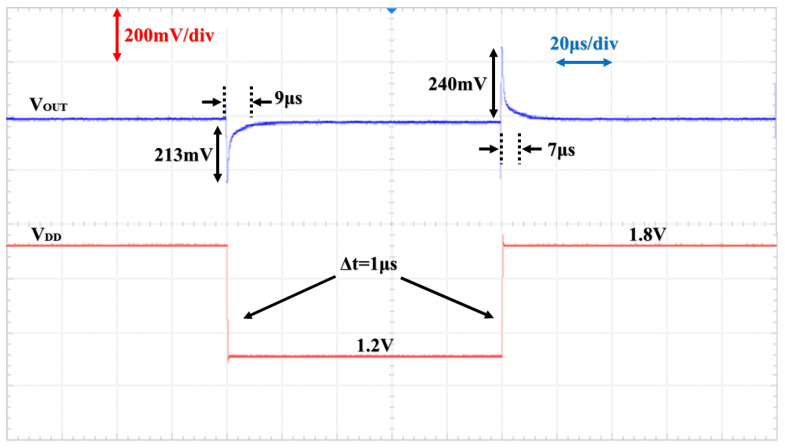
Line transient response as $I_{\mathrm{Load}}$ = 10 μA.

With a load current of 10 μA, changing the input voltage from 1.2 V to 1.8 V in 1 μs brings about an overshoot voltage of 240 mV and takes 7 μs to recover to the designed output voltage. And when the input voltage is reduced, the undershoot voltage is 213 mV, for a recovery time of 9 μs as shown in Figure 18.

**Figure 19:**
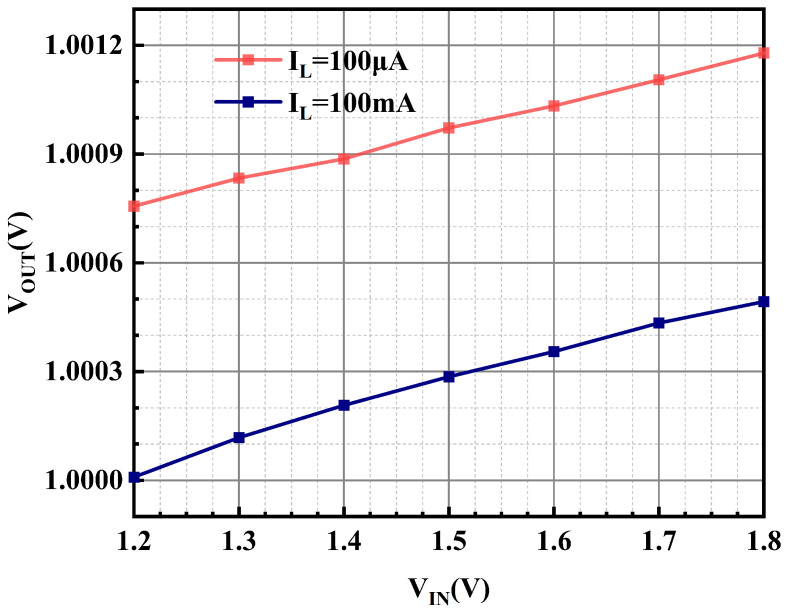
Measured line regulation of the proposed OCL-LDO under different loads.

The low dropout regulator is a circuit structure that is capable of delivering a stable voltage value that has no significant deviation with the input voltage variation. In order to feature the effect of input voltage variation on the output voltage, the line regulation is proposed; Figure 19 shows the measurement results of the OCL-LDO proposed in this paper for the variation in the output voltage with the input voltage under different load currents, and its line regulation is 0.7 mV/V and 0.87 mV/V for load currents of 100 μA and 100 mA.

**Figure 20:**
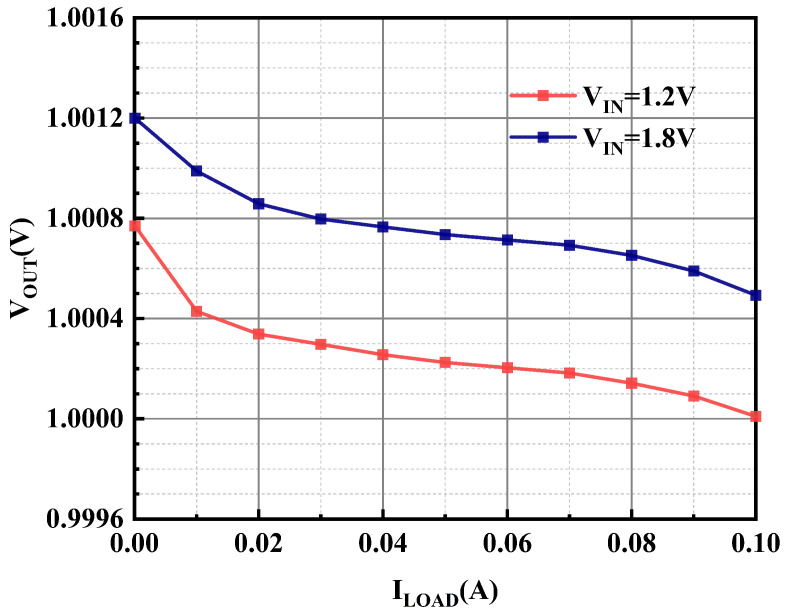
Measured load regulation of proposed OCL-LDO under different supply voltages.

With similarity, the variation in the load current is also not supposed to make the output voltage generate large fluctuations. The experimental results of the load regulation of the OCL-LDO proposed in this paper are shown in Figure 20, which are 7.6 μV/mA and 7.3 μV/mA when the output voltage is 1 V, and when the input voltage is 1.2 V and 1.8 V, respectively.

**Figure 21:**
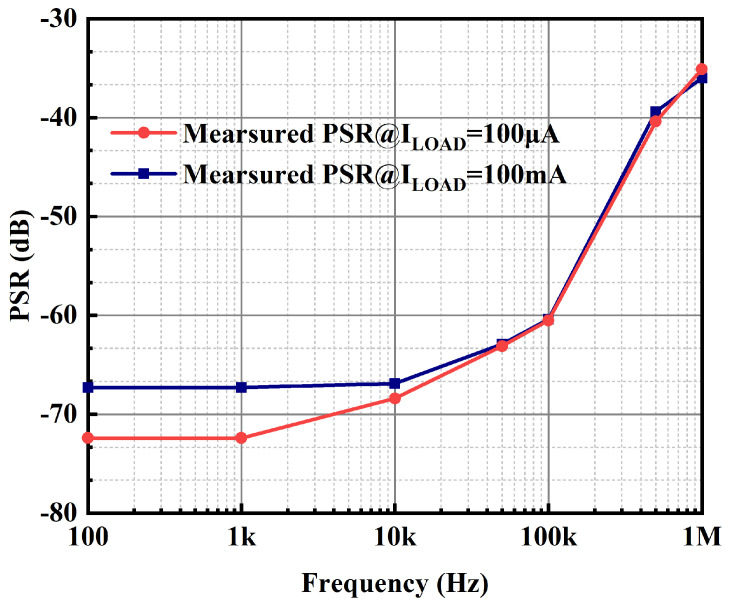
Measured PSR of the proposed OCL-LDO.

The OCL-LDO is required to have favorable ripple immunity to provide a stable voltage supply, so its PSR performance is also extremely important. The PSR test results of the OCL-LDO proposed in this paper are provided in Figure 21. Since the adaptive biasing scheme is included within the OCL-LDO proposed in this paper, the bias current of the error amplifier increases when the load current is high. The primary pole of the error amplifier increases, which also results in the PSR of the OCL-LDO having a larger zero at heavy loads than the corresponding zero at low loads [31]. The PSR at 1 KHz is −72.4 dB when the load current is 100 μA.

**Figure 22:**
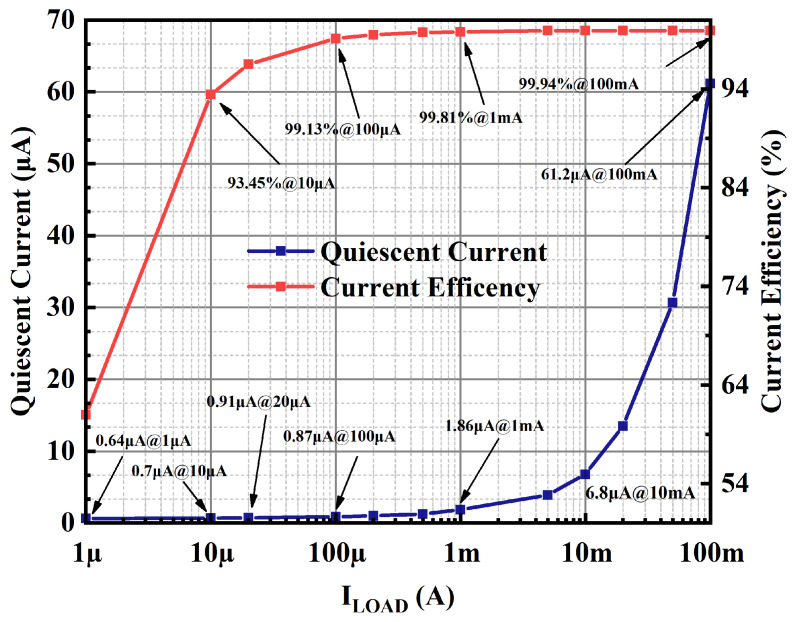
Measurement results of quiescent current and current efficiency.

The quiescent current of this OCL-LDO increases with the load currents because of the adaptive biasing scheme within the OCL-LDO proposed in this paper. Measurement of the quiescent current of the OCL-LDO is performed by utilizing the test circuit schematic shown in Figure 15, which requires the supply voltage that is 1.2 V to be provided by the $V_{\mathrm{DC}}$, and the input voltage is 1 V. The load currents could be calculated according to the different load resistances, and the quiescent current of the OCL-LDO is obtained by subtracting the load current from the current flowing out of the power supply. The quiescent current of the OCL-LDO proposed in this paper varies from 0.64–61.2 μA as shown in Figure 22. Meanwhile, the current efficiency characterizes the effective conversion rate of the OCL-LDO to the input signals, and the peak current efficiency of the OCL-LDO proposed in this paper is 99.94%.

## 5. Discussion

For comprehensive characterization of the performance of proposed OCL-LDO, a Factor of Merit (FOM) is presented, and its expression is given as [32]:

(14) $$FOM=\frac{\Delta V_{out}\times C_{L}\times I_{Q}}{I_{L,\max}^{2}}$$

where Δ$V_{out}$ is the sum of the overshoot and undershoot voltages, $I_{Q}$ is the quiescent current, and $I_{L,max}$ is the maximum load current. The key performance results of the OCL-LDO proposed in this paper and its comparison with prior works are provided in Table 2. As seen in the table, the OCL-LDO proposed in this paper is suitable for NB-IoT applications with good measurement results of the transient response, PSR, line regulation, and load regulation based on a low quiescent current.

## 6. Conclusions

An ultra-low quiescent current OCL-LDO for narrow band IoT applications is proposed in this paper. In this paper, its overall structure and stability analysis are introduced, and the small-signal transfer function with and without considering the slew-rate enhancement circuit is given respectively. Meanwhile, the specific circuit design and key design considerations are shown in this paper, comparing the simulation results of the error amplifier before and after the addition of the gain increase method, and proposing a sub-threshold CMOS slew-rate enhancement circuit. The experimental results show that the proposed OCL-LDO has a minimum quiescent current of 640 nA and a maximum load current of 100 mA when the input voltage is 1.2 V and the output voltage is 1 V. The overshoot and undershoot voltages when the load current is switched between 100 μA and 100 mA are 197 mV and 201 mV, respectively, and both of them have a recovery time of less than 2 μs. Therefore, the OCL-LDO proposed in this paper features a favorable transient response based on an nA-level quiescent current, which is suitable for low-power NB-IoT applications.

## Figures and Tables

**Table 1:** Comparison of simulated results and calculated results of DC gain.

	Calculated Result	Simulation Result
Without NR and CCIS	73.6 dB	72.1 dB
With NR and without CCIS	77.9 dB	75.9 dB
With NR and CCIS	82.4 dB	81.6 dB

NR: negative resistance. CCIS: cross-coupled inputs stage.

**Table 2:** Performance summary and comparison with prior works.

References	[8] ^*m*^	[10] ^*m*^	[18] ^*m*^	[19] ^*m*^	[21] ^*s*^	[26] ^*s*^	This Work ^*m*^
Year	2018	2024	2024	2022	2024	2023	2024
Technology [nm]	180	28	500	180	180	180	180
$V_{\mathrm{IN}}$ [V]	1.2–1.25	0.4–1.1	2.8–5.0	0.6-1	1.2–1.8	0.6	1.2–1.8
$V_{\mathrm{DROP}}$ [V]	0.2	0.2	0.2	0.1	0.2	0.1	0.2
$V_{\mathrm{OUT}}$ [V]	1	0.2–1.05	1.4–4.8	0.5	1	0.5	1
$C_{L,\max}$ [pF]	100	80	100	N/A	100	1000	100
$I_{Q,\min}$ [µA]	0.407	0.6	33	0.007	47	0.22	0.64
$I_{L}$ [mA]	0–100	1–165	1–300	0.01–0.75	0–20	0.01–10	0.001–100
Peak η [%]	99.75	99.98	N/A	99.96	99.99	99.96	99.94
Line Reg. [mV/V]	0.283	1	0.1	3.18	1.5	0.487	0.87
Load Reg. [µV/mA]	77	60	40	4770	25	0.00585	7.6
Δ$I_{L}$/Δt [mA/ns]	100/300	148/10	299/50	0.49/N/A	20/100	9.99/1	99.9/500
Overshoot [mV]	35.33	50	182	N/A	156	99	197
Undershoot [mV]	117	47	276	N/A	238.6	230	201
PSR [dB]	−46.82@10k	−44.3@100k	−58@10k	−22.5@10	−43@100	−43.97@1k	−72.4@1k
FOM [fs]	0.62	39	16.94	N/A	4636.55	723.8	2.55
Active area [mm^2^]	0.055	0.023	0.13	0.043	0.0105	N/A	0.049

^*m*^: Measurement results. ^*s*^: Simulation results.

## Data Availability

The original contributions presented in the study are included in the article, further inquiries can be directed to the corresponding author.
